# Primase-polymerases are a functionally diverse superfamily of replication and repair enzymes

**DOI:** 10.1093/nar/gkv625

**Published:** 2015-06-24

**Authors:** Thomas A. Guilliam, Benjamin A. Keen, Nigel C. Brissett, Aidan J. Doherty

**Affiliations:** Genome Damage and Stability Centre, School of Life Sciences, University of Sussex, Brighton BN1 9RQ, UK

## Abstract

Until relatively recently, DNA primases were viewed simply as a class of proteins that synthesize short RNA primers requisite for the initiation of DNA replication. However, recent studies have shown that this perception of the limited activities associated with these diverse enzymes can no longer be justified. Numerous examples can now be cited demonstrating how the term ‘DNA primase’ only describes a very narrow subset of these nucleotidyltransferases, with the vast majority fulfilling multifunctional roles from DNA replication to damage tolerance and repair. This article focuses on the archaeo-eukaryotic primase (AEP) superfamily, drawing on recently characterized examples from all domains of life to highlight the functionally diverse pathways in which these enzymes are employed. The broad origins, functionalities and enzymatic capabilities of AEPs emphasizes their previous functional misannotation and supports the necessity for a reclassification of these enzymes under a category called primase-polymerases within the wider functional grouping of polymerases. Importantly, the repositioning of AEPs in this way better recognizes their broader roles in DNA metabolism and encourages the discovery of additional functions for these enzymes, aside from those highlighted here.

## INTRODUCTION

Replicative DNA polymerases are exquisitely fine-tuned to synthesize DNA in a highly accurate and efficient manner, however, they are unable to initiate DNA synthesis *de novo*. As a consequence, DNA replication requires an initiating step to generate a primer containing the essential 3′ hydroxyl moiety that is requisite for polymerase-dependent synthesis. Early studies identified that this ‘priming’ role is fulfilled by specialized RNA polymerases capable of synthesizing short RNA chains, generating primers that enable DNA synthesis to be initiated (1,2). Subsequent studies revealed that this role is fulfilled by specialized DNA-dependant RNA polymerases, distinct from classical RNA polymerases. These ubiquitous and indispensable enzymes were named DNA primases (3,4).

Due to the semi-discontinuous nature of DNA replication, primases are essential, not only during initiation, but also throughout the process of lagging strand replication, where they initiate synthesis of the discontinuously synthesized Okazaki fragments. However, the activities and cellular roles of these enzymes extends further than this essential role in initiating DNA replication. Over recent years, significant evidence has accumulated suggesting that primases should no longer be viewed simply as replication initiation-specific DNA-dependant RNA polymerases. Instead, members of this superfamily comprise a functionally diverse group of ancient enzymes that undertake a wider variety of cellular roles in DNA replication, repair and, possibly, also in transcription. Drawing on examples from all domains of life, with a particular focus on the archaeo-eukaryotic primases (AEPs), this review will explore and highlight the diverse roles of these enzymes that reposition them as a multi-faceted group of polymerases required for a wide variety of cellular roles including replication, repair and damage tolerance, in addition to primer synthesis.

## TWO DISTINCT PRIMASES: DnaG AND AEP PRIMASE SUPERFAMILIES

Early studies identified that the *Escherichia coli* DnaG protein is responsible for the initiation of Okazaki fragment synthesis by the generation of short RNA primers (5). All bacteria possess DNA primases belonging to the DnaG superfamily, which fulfil the canonical primer synthesis role during DNA replication. Typically, these monomeric DnaG-like replicative primases are helicase-associated, permitting the synthesis of RNA primers of between 10 and 60 nt in length on most ssDNAs (6). These enzymes contain a characteristic catalytic domain of the topoisomerase-primase (TOPRIM) fold, composed of an α/β core with four conserved strands and three helices, in addition to two conserved catalytic motifs (7). The first of these motifs, containing a conserved glutamate, is thought to act as a general base during nucleotide polymerization. The second motif contains two conserved aspartates (DxD), which coordinate Mg^2+^ ions required for catalytic activity (8).

**Figure 1.**
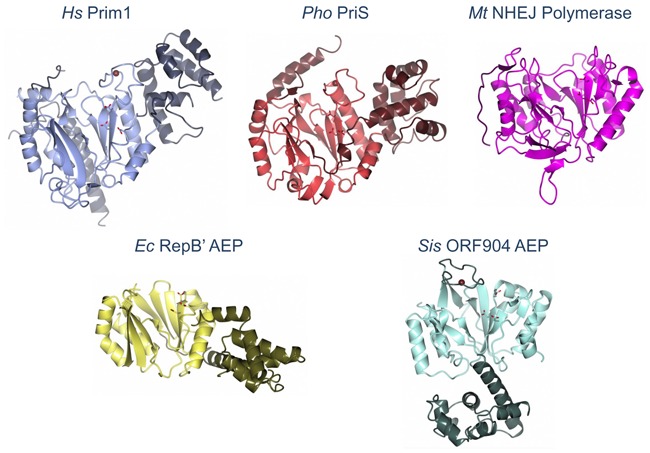
Architecture of AEP catalytic subunits from the major domains of life. Representative examples of the crystal structures of AEPs that have been elucidated. Human primase subunit (Prim1) (PDBID: 4RR2), sky blue. The primase small catalytic subunit (PriS/Prim1) from the archaeal species *Pyrococcus horikoshi* (PDBID: 1V33), pale crimson. The NHEJ repair polymerase (PolDom/LigD-Pol) from *Mycobacterium tuberculosis* (PDBID: 2IRU), magenta. The AEP domain of RepB’ encoded by the *Escherichia coli* plasmid RSF1010 (PDBID: 3H20), gold. The AEP domain of ORF904 encoded by the *Sulfolobus islandicus* plasmid pRN1 (PDBID: 3M1M), sea green. The conserved catalytic core of these enzymes is shown in a lighter hue and catalytic triads are rendered as sticks with the acidic oxygens coloured red. Where present, the coordinated zinc atoms in the zinc finger domains are coloured tan.

**Figure 2.**
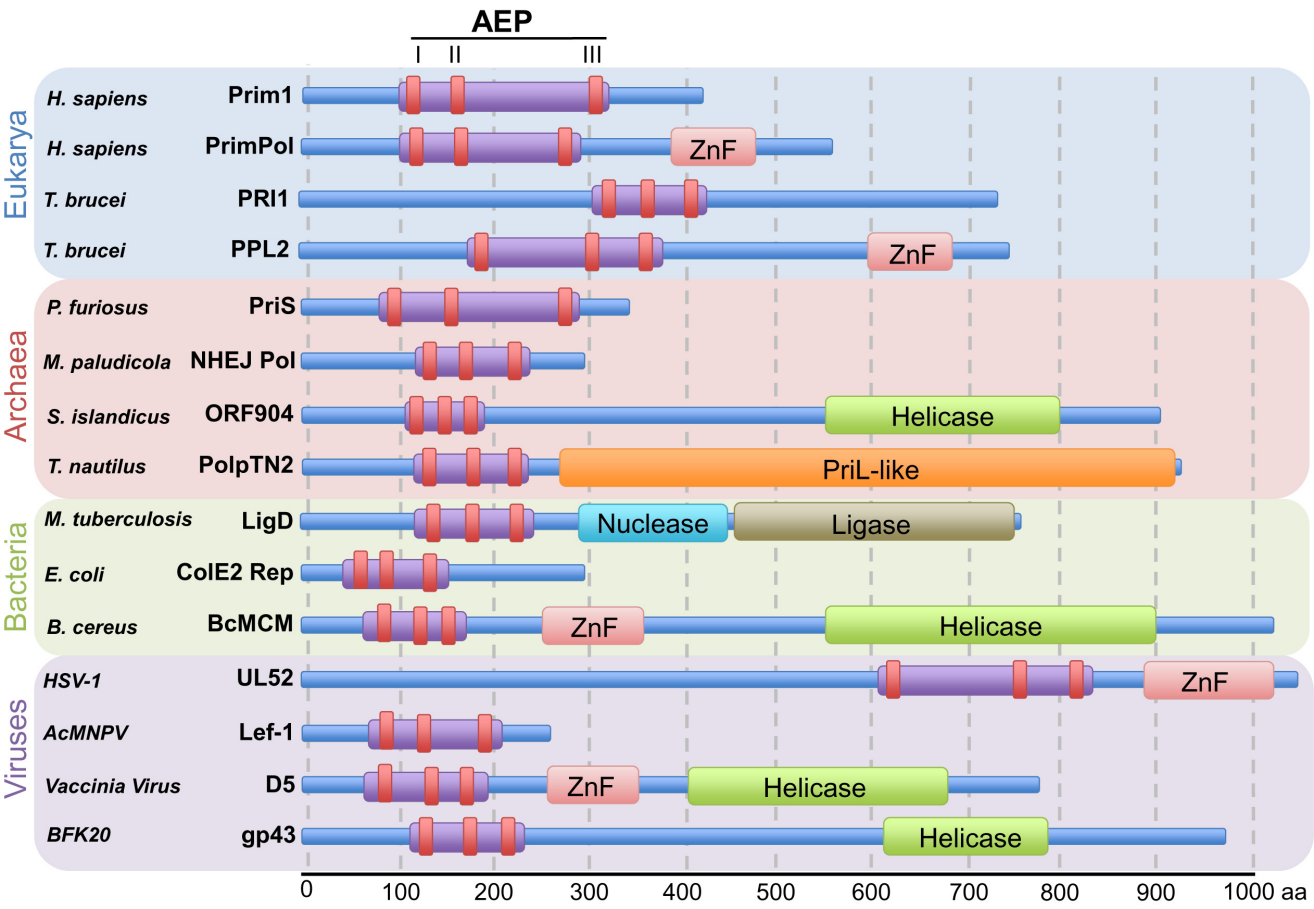
Domain organization of members of the AEP superfamily. The AEP superfamily is formed of a number of divergent enzymes with varying domain organizations. Some representative examples of members of the AEP superfamily are displayed here with the three signature catalytic motifs of the AEPs depicted in red, in addition to any accessory domains associated with this domain. The blue, red, green and purple backgrounds correspond to the different domains of life to which these primase family members belong.

Although functionally related, the AEP superfamily is evolutionarily and structurally distinct from the bacterial DnaG primases (6–10). In common with prokaryotic DnaG primases, AEPs are absolutely required for the initiation of DNA replication in archaea and eukaryotes (6). Despite this, DnaG-like primases have been identified in archaeal genomes and, similarly, AEPs are also found to be distributed across all domains of life. Replicative primases of the AEP superfamily typically form a heterodimeric complex containing both a small catalytic subunit (PriS/Prim1) and a large accessory subunit (PriL). In eukaryotes, this heterodimer forms a complex with the DNA Pol α subunits (A and B) that together initiate DNA replication (6). The short RNA primer synthesized by the primase subunit is elongated by Pol α, a member of the Pol A family, to generate longer DNA primers that are subsequently extended by the replicative DNA Pols ϵ and δ. The AEP superfamily is distinguished by a characteristic catalytic core composed of two modules; an N-terminal (αβ)_2_ unit that has no equivalent structural homology to other proteins in the structural database (PDB) and a C-terminal unit, which like the A- B- and Y-family DNA polymerases, is a highly derived RNA recognition motif (RRM) (Figure 1). This catalytic core harbours three conserved motifs (motifs I, II and III), an hhhDhD/E motif (where ‘h’ is a hydrophobic residue), an sxH motif (where ‘s’ is a small residue and ‘x’ is any residue) and an hD/E motif (11). The first and third of these motifs are involved in divalent metal ion coordination for catalysis, whilst the sxH motif is required for nucleotide binding (10,12–13). Multiple mutagenesis studies have shown these motifs to be essential for catalysis (10,14–21). In addition to these motifs, some AEPs also possess additional associated domains including zinc-binding and helicase domains (Figure 2).

Despite the apparent uniqueness of the AEP catalytic fold, the highly conserved catalytic aspartate residues of these enzymes are superposable with the catalytic core of the X-family DNA polymerases, including Pol β (10,22). However, this apparent similarity is thought to be a result of convergent evolution as the secondary structural contexts in which these aspartate residues are located differs (12,23). Nevertheless, this similarity, coupled with the requirement of divalent metal ions for catalysis, allows inference of a two-metal ion mechanism of catalysis, similar to that employed by DNA polymerases (22,24). This mechanism is now supported by the structure of a pre-ternary complex of a mycobacterial AEP bound to DNA (25), see below for details.

## EVOLUTIONARY HISTORY OF AEPs

**Figure 3.**
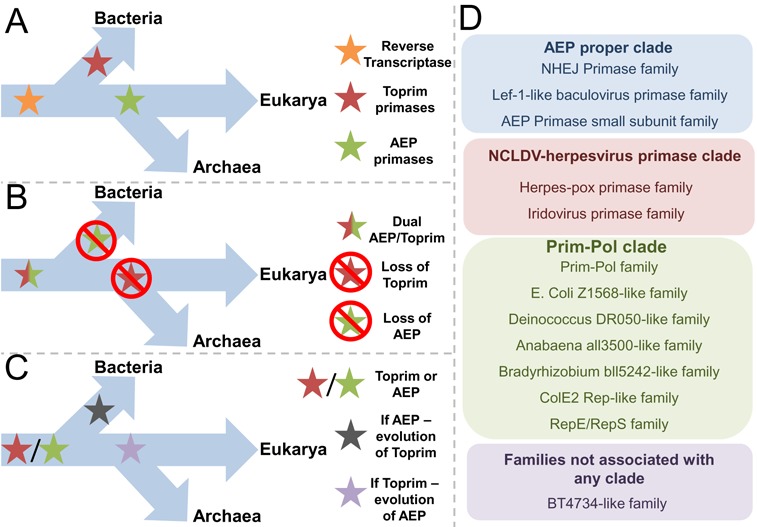
Alternative hypotheses for the evolution of AEP and Toprim primases. (**A**) The first model of primase evolution suggests that primases evolved independently twice from a last universal common ancestor (LUCA). Bacterial ancestors evolved toprim primases and archaeal and eukaryotic ancestors evolved AEP primases. Subsequent horizontal gene transfer occurred between the two lineages to account for AEP primase's role in NHEJ in bacteria and toprim-type primase's role in archaeal RNA degradation. (**B**) The second model of primase evolution suggests that LUCA had a dual primase replication mechanism, consisting of both AEP and toprim primases. During the evolution of bacteria, they lost the replicative function of the AEP primases but retained them for the auxiliary function of NHEJ-mediated DNA repair. During the evolution of the archaeal/eukaryotic lineage, the replicative function of toprim primases was lost but their auxiliary role in archaeal RNA degradation was retained. (**C**) The third model of primase evolution suggests that LUCA had either an AEP or toprim-like primase. Significant, evolutionary pressures could then have driven the evolution or acquisition of a second class of primase. (**D**) 12 of the 13 major AEP families can be arranged into three higher order clades, the AEP Proper Clade, the NCLDV-Herpesvirus primase clade and the Prim-Pol clade.

The lack of homology between the bacterial and archaeal/eukaryotic primase superfamilies also extends to other replicative proteins of these domains, including DNA polymerases and helicases. This clear distinction between the replicative machinery employed by bacteria and archaea/eukaryotes has generated debate as to how these two groups of replicative enzymes arose. Notably, in contrast to the differences in DNA replication machinery, the core components of transcription and translation are conserved across domains (26,27). This observation led to a hypothesis that the two replicative systems evolved twice independently from a common ancestor which utilized reverse transcription to replicate an RNA/DNA genome (27) (Figure 3A). The evolution of DNA replication-competent cells then subsequently led to the elimination of the reverse transcription pathway. In support of this model, a number of primases and polymerases possess, or can be engineered to exhibit, reverse transcriptase activity (28–32). A second model proposes that the last universal common ancestor (LUCA) possessed both an AEP and TOPRIM primase (33) (Figure 3B). In bacteria, selective pressure resulted in the loss of AEPs as replicative primases and, similarly, in archaea TOPRIM primases lost their role in priming replication. Importantly, many bacteria and archaea still retained their respective AEP and TOPRIM primases, however the roles of these enzymes changed with the AEPs employed in DNA repair processes, e.g. non homologous end-joining (NHEJ) in bacteria (16) and the TOPRIM primases potentially utilized for RNA degradation in archaea (34,35). This model also predicts that in eukarya the DnaG primase was lost and other proteins were acquired to replace its roles (33). An alternative scenario to these models is that LUCA possessed either a TOPRIM primase or an AEP and subsequent selective pressure led to the emergence of the second primase superfamily in either the bacterial or archaeal/eukaryotic lineages (Figure 3C). In this case, AEPs could have been acquired later by bacteria and viruses through horizontal gene transfer to fulfil alternative roles in DNA replication, repair and damage tolerance. Which of these models is likely to be correct remains to be established.

Despite the lack of homology between the primase superfamilies, the evolutionary history of the AEP superfamily displays an interesting parallel with that of the DnaG TOPRIM primases. Iyer *et al*. reported an extensive *in silico* analyses of the AEP superfamily and identified that the closest relatives of the AEP-fold, amongst the RRM-like proteins, are the rolling circle replication endonucleases (RCRE) and origin-binding domain proteins (OBDs) of the papovaviruses. This close evolutionary relationship between the AEPs and RCRE echoes that of the DnaG TOPRIM primases with topoisomerases. Intriguingly, this reveals that both primase superfamilies share close evolutionary ties with nucleases, which offer an alternative solution to the DNA replication initiation problem (11). Specifically, transfer of the 5′ end of a nicked DNA strand to a tyrosine on the nuclease allows the elongation of the free 3′ OH group by a DNA polymerase for synthesis of the new strand. In several families of DNA viruses and phage, this method of initiating rolling circle replication is employed. Iyer *et al*. suggest that RCRE and OBDs share a common ancestor with the AEPs that possessed polymerase activity. The RCRE subsequently evolved from this enzyme by acquiring nuclease and losing polymerase activities, meanwhile OBDs lost all catalytic activity. However, the authors also accept the possibility that the AEP-RCRE-OBD common ancestor was simply a nucleic acid binding protein, which utilized its divalent cation coordinating acidic residue to aid in DNA binding. This ancestral protein may then have acquired nuclease activity, whilst polymerase activity could have been independently acquired in numerous descendent lineages (11).

To date, the AEP superfamily can be classified into 13 major families, 12 of these can be further organized into three higher-order clades; the AEP proper clade, the NCLDV-herpesvirus clade and the PrimPol clade (Figure 3D) (11). Regardless of the somewhat murky evolutionary origins of the AEP superfamily, studies in recent years have illustrated that these enzymes have diversified to fulfil a range of specialist roles in DNA replication, repair and damage tolerance, as will be described below.

## ARCHAEAL PRIMASES CAN ACT AS REPLICATIVE POLYMERASES

**Figure 4.**
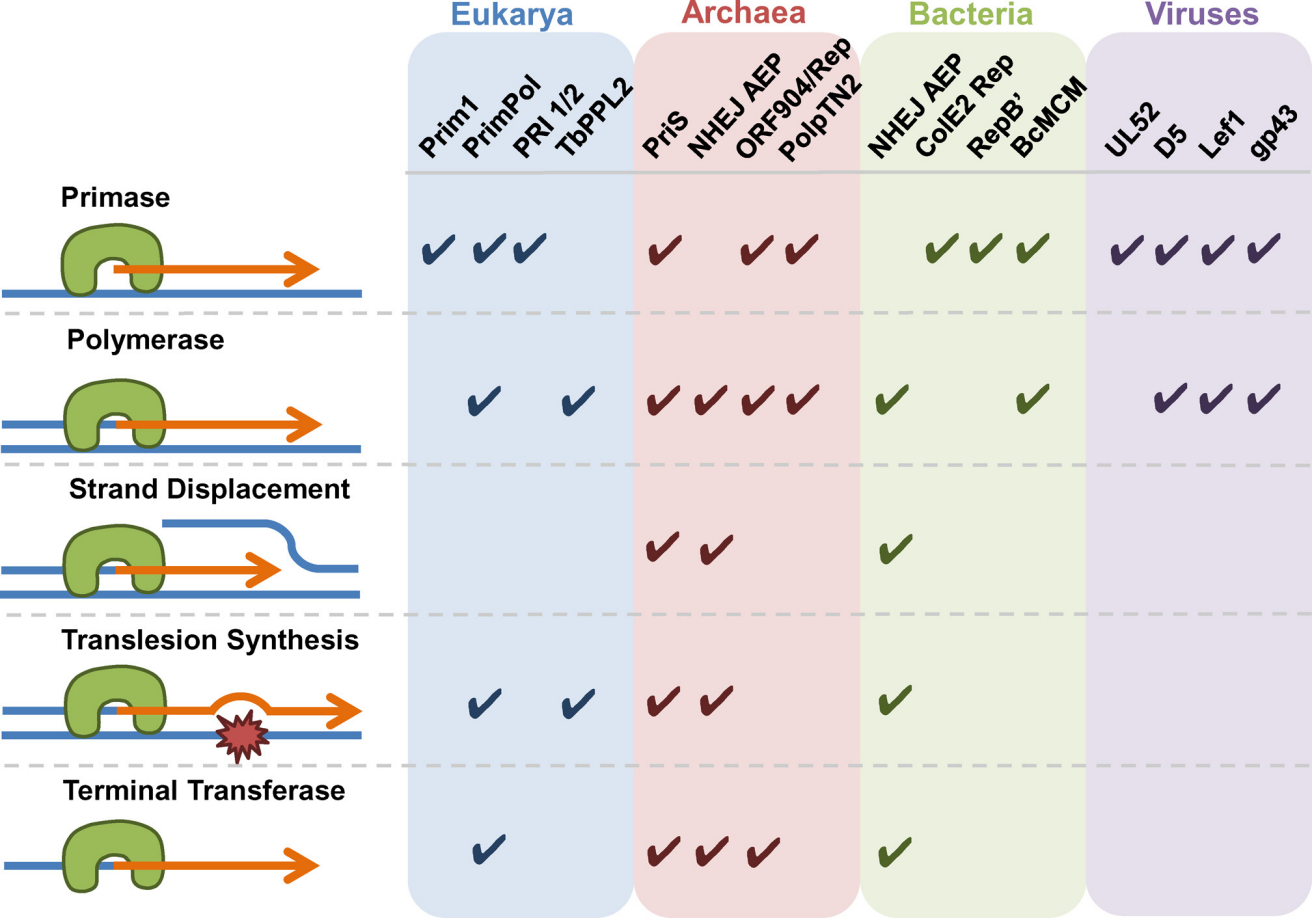
Nucleotidyltransferase activities associated with AEP members. AEP-type primase family members possess many more activities, in addition to catalyzing primer synthesis DNA for replication. The reported additional nucleotidyltransferase activities for each of the different AEPs are depicted, including polymerase activity (either DNA-dependent DNA polymerase or DNA dependent RNA polymerase), lesion bypass, terminal transferase and strand-displacement. The observed ability of each enzyme to perform the indicated activity is noted by a tick. The blue, red, green and purple backgrounds correspond to the domain of life in which the primase family is found.

In eukaryotes, the replicative heterodimeric primase (PriS/L or Prim1/2) complexes with Pol α (A/B) to form a tetrameric complex. The resolution of the crystal structure of the human heterodimeric primase identified that the small subunit (Prim1) utilizes the same set of functional residues for primer initiation and elongation (Figure 1), in addition, this study also identified the mode of association between the primase and Pol α (36,37). A more recent study of the intact full-length primase noted the presence of a long linker region between the N- and C-terminal domains of the p58 subunit, required for initiation and elongation during primer synthesis as well as the enzyme's conformational flexibility (38). The partnership between the eukaryotic primase and Pol α allows the PolA subunit to extend short RNA primers synthesized by the primase subunits, before handing over synthesis to the more processive and accurate replicases (Pols ϵ and δ). However, this complexity does not exist in archaea, which lack Pol α subunits (A or B) or any apparent interaction between the PriS/L complex and the archaeal family-B replicases (34,39). Remarkably, the replicative primase is able to synthesize and elongate its own primers. Evidence for archaeal primases as DNA-dependent DNA polymerases was first noted in the archaeal Prim1 homologue, PriS, from the hyperthermophile *Pyrococcus furiosus* (*Pfu*) (40). In addition to displaying DNA polymerase activities (Figure 4), *Pfu* PriS can also synthesize primers using dNTPs, as well as NTPs. This contrasts with eukaryotic Prim1, which can only synthesize RNA primers (39). This ability to synthesize DNA primers and perform DNA-dependent DNA polymerase activity has also been observed in the PriS/L complexes from other archaea, including *Pyrococcus horikoshii* (41), *Sulfolobus solfactaricus* (19), *Thermococcus kodakaraensis* (17) and *Archaeoglobus fulgidus* (32). In fact, for each of these species, except *T. kodakaraensis*, the replicative primase actually shows a preference to prime using dNTPs over NTPs. Strikingly, the primer elongation capacity of these enzymes ranges from less than 500 bases in length to >7 kb (17,19,34,39–40). Together, these studies provided the first evidence that archaeal replicative primases can also be utilized in a role similar to that of Pol α in eukaryotes, thus establishing that these enzymes can act and be classified as primase-polymerases (Prim-Pols).

## PRIMASES INVOLVED IN DNA DOUBLE-STRAND BREAK REPAIR

Around the time that archaeal primases were first reported to also be template-dependent polymerases (40), AEP orthologues were unexpectedly identified in prokaryotic genomes (9,42–43). These AEP genes were frequently found to be co-operonic with Ku (9,43–44), a protein responsible for binding the ends of DNA double-strand breaks (DSBs) during NHEJ repair in eukaryotes. These findings provided early clues that a conserved NHEJ pathway may exist in prokaryotes and that AEPs may be intrinsically involved in this DSB repair process. Subsequent studies identified that a *bona fide* NHEJ DSB repair apparatus exists in bacteria (16,45–47) and that these AEPs form part of a larger multi-protein repair complex known as ligase D (LigD). More recently, a closely related NHEJ apparatus has also been identified in some archaeal species (48). In mycobacteria, LigD is a fusion protein composed of AEP, nuclease and ligase domains (16,49). However, in many species these ‘domains’ exist as individual co-operonically expressed proteins that form a functional NHEJ complex (48). Prokaryotic NHEJ is therefore thought to be essentially facilitated by a Ku–LigD complex that possesses all of the activities required to bind to the break termini and catalyze re-joining of DSBs (16,46–48). NHEJ AEPs are capable of performing an astonishing range of nucleotidyltransferase activities, presumably to accommodate the myriad of end configurations produced during formation of DSBs. Specifically, these enzymes can catalyze template-dependent DNA/RNA polymerase, terminal transferase, strand-displacement and gap-filling synthesis, with a notable preference to incorporate ribonucleotides (16,46,48). In addition, these AEPs can readily extend mismatched primer-template termini and perform translesion synthesis (TLS) bypass of 8-oxo-2’-deoxyguanosine (8-oxo-dG) lesions and abasic sites (46) (Figure 4).

**Figure 5.**
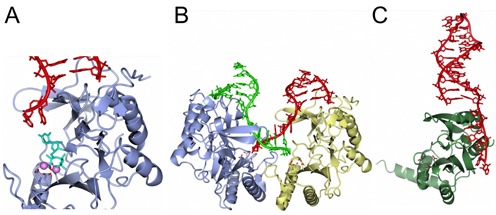
Structures of AEPs bound to DNA substrates. Structural examples of AEP members bound to DNA intermediates. (**A**) Structure a pre-ternary catalytic conformation of a NHEJ repair polymerase (PolDom/LigD-Pol) from *Mycobacterium tuberculosis* bound to a ds DNA break with a 3′ overhanging terminus (PDBID: 4PKY, ice blue) with UTP and manganese cofactors, coloured cyan and pink, respectively. (**B**) Crystal structure of a micro-homology mediated end-joining (MMEJ) intermediate showing an NHEJ repair polymerase-mediated synapsis of a DSB (PDBID: 4MKY, ice blue and lemon). (**C**) Structure of the AEP domain of RepB’ bound to a *ssiA* DNA replication initiation site (PDBID: 3H25, lawn green). The catalytic residues are rendered as sticks with the acidic oxygens coloured red. DNA strands are coloured red or green.

Since the unexpected discovery that AEPs function as components of the DSB repair machinery in bacteria and archaea, there has been much conjecture about why members of the primase family evolved to become the primary NHEJ polymerases. It is likely that these bespoke repair enzymes, which belong to the AEP proper clade that also includes the replicative primases, evolved from a primordial AEP with an innate capacity to make short RNA primers into a novel class of adaptable end-joining polymerases capable of processing DNA ends during break repair. A comparison of the sequences and structures of the NHEJ AEPs with the replicative enzymes (PriS), reveals that whilst both share a common catalytic architecture (Figure 1), there are several distinctive adaptations. NHEJ AEP polymerases possess a number of distinctive DNA binding modes that distinguishes them from related enzymes, enabling them to operate even at the extreme ends of DNA. These enzymes possess a positively charged surface pocket that enables them to bind specifically to a 5′ phosphate, either close to or at the terminus of a DSB, thus stably tethering the enzyme to the break to permit end-processing (Figure 5A). In addition, they have also evolved prominent surface loops (Loops 1 and 2) that facilitate a remarkable ability to promote break synapsis (Figure 5B), a process that permits breaks to be annealed back together by a mechanism known as microhomology-mediated end joining (MMEJ). During this process, each side of the break is first bound by a polymerase, forming a pre-ternary complex in anticipation of receiving the other end of the break. Subsequently, the surface loops, conserved only in these AEPs, act in concert to ‘present’ one end of the DSB to the other side in order to promote and accelerate break annealing. In the case of the 3′ overhangs, this process configures the break to allow productive gap-filling synthesis to occur *in trans* (25,50–51). This mechanism also provides a molecular basis for the template-dependent terminal transferase synthesis catalyzed by these enzymes at the 3′ ends of DNA. Although these unprecedented MMEJ processes were initially considered by some to be specific to these polymerases, an analogous polymerase-mediated MMEJ mechanism has since been reported for archaeal PriS, mammalian Pol θ and terminal transferase (TdT), suggesting that this is a functionally conserved mechanism (33,52–53).

Although the biological roles of the NHEJ and replicative AEPs are clearly distinct, these enzymes are closely related, belonging to the same clade and therefore are likely to share common features despite their divergent evolution. The crystal structures of a number of catalytic intermediates of mycobacterial NHEJ AEPs bound to DNA have provided some important insights into the shared common catalytic mechanisms of AEPs and also explained why these enzymes may be suited to the task of break repair (25,50–51). The structure of a pre-ternary NHEJ AEP–DNA complex has revealed that these enzymes, in common with polymerases, employ a two metal mechanism of catalysis, with binding of the second metal dependent on engagement of the incoming nucleotide with both the active site and template strand (Figure 5A). As discussed, these AEPs have the ability to ‘accept’ an incoming primer strand provided *in trans* by an adjacent AEP pre-ternary complex (Figure 5B). Significant in this regard, these enzymes can bind to and extend an incoming primer as short as a dinucleotide (25). This mechanism is highly reminiscent of the initiation step performed by replicative primases. Here, a binary complex between the enzyme and ssDNA is formed first, followed by binding of the 3′ nt to form a pre-ternary complex. This is followed by recruitment of a 5′-nt, which acts as the ‘primer’, to form a ternary complex. Notably, both NHEJ and replicative AEPs can catalyse an unconventional addition of a ribonucleotide in the 3′-5′ direction, followed by a more conventional 5′-3′ elongation step. This innate ability of AEPs of this clade to accept short primers may explain why they were the most appropriate enzymes to evolve further, by the acquisition of additional surface loops and phosphate binding residues, into highly effective NHEJ repair polymerases.

## PRIMASES INVOLVED IN DNA DAMAGE TOLERANCE

In addition to the apparent absence of Pol α homologues, many archaeal species also lack canonical TLS polymerases, required to bypass replicase stalling lesions, with only a subset of species possessing Y-family TLS polymerases. Furthermore, many archaea do not encode canonical nucleotide excision repair or photolyase pathways to remove potential replication fork stalling UV-light induced DNA damage (54). This raises the question as to how archaeal species lacking these pathways tolerate DNA damage, which is of paramount importance given the extreme environments in which many of these species reside. Recently, it was reported the replicative primase, PriS, from Y-family deficient archaeal species (*A. fulgidus* and *P. furiosus)* is inherently damage tolerant (Figure 4) (32). Strikingly, it was identified that PriS from these organisms is capable of faithfully bypassing the highly DNA-distorting UV-induced lesions cyclobutane pyrimidine dimers, in addition to bypass of 8-oxo-dG, a product of oxidative DNA damage. The extreme environments inhabited by thermophilic archaeal species generate significant amounts of cytosine deamination, generating uracil base adducts that induce profound fork stalling when encountered by the archaeal replicases (B- and D-family Pols). Notably, PriS also replicates past templating uracil bases, even when stalled replicative polymerases are bound (32), suggesting that PriS assists the replisome in maintaining active fork progression during genome duplication. These findings further corroborate that archaeal replicative primases, in addition to primer synthesis, play additional roles in replication.

Until recently, the only AEP member identified in higher eukaryotes was the Pol α-associated, PriS homologue, Prim1. However, a second eukaryotic AEP has now been described and characterized, this enzyme is named PrimPol (alternative names CCDC111, FLJ33167, EukPrim2 or hPrimPol1). PrimPol was originally identified as a novel uncharacterized member of the NCLDV-herpes virus clade of AEPs (11). PrimPol orthologues are present across a diverse range of unicellular and multicellular eukaryotes including species of animals, plants, and primitive early eukaryotes, such as fungi, protists and algae. However, PrimPol is notably absent from a number of eukaryotes including *Caenorhabditis elegans* and *Drosophila melanogaster*. Importantly, this interrupted distribution suggests that PrimPol was acquired through horizontal gene transfer from a viral source and subsequently lost on a number of independent occasions (11). The enzyme is composed of two domains, an N-terminal AEP domain, consisting of three canonical catalytic motifs, and a C-terminal UL52-like zinc finger conserved across the NCLDV-herpesvirus clade of AEPs (Figure 1) (11). PrimPol, similar to *A. fulgidus* and *P. furiosus* PriS, possesses both primase and DNA damage tolerance polymerase activities (Figure 4) (14,55–59). Specifically, PrimPol is capable of bypassing UV-induced 6–4 photoproducts, in addition to 8-oxo-dG lesions (14,55). In common with canonical TLS polymerases, PrimPol exhibits low fidelity, very limited processivity (1–4 bases) and an error-prone specificity that is highly biased towards insertion-deletion (indel) errors (60).

PrimPol is required for the maintenance of replication fork progression and PrimPol knockout cells display decreased forks rates and increased sensitivity to DNA damaging agents (14). Furthermore, PrimPol has been implicated in the restart of stalled replication forks (56–57,59), involving either translesion synthesis or re-priming downstream of damage. Unlike canonical TLS polymerases, PrimPol appears to be recruited to stalled forks through interactions with the single-strand binding protein Replication Protein A (RPA), rather than through mono-ubiquitinated PCNA (59,60). Intriguingly, this interaction also serves to limit the potentially mutagenic contribution of PrimPol to DNA synthesis by restricting both the primase and polymerase activities of the enzyme (60). Similarly, RPA has also been shown to strongly inhibit the primase activity of Prim1/2 to prevent non-specific priming events, an effect which is relieved by the SV40 T antigen helicase (61). Low fidelity synthesis has also been noted in other AEPs and it remains possible that similar regulatory mechanisms exist to restrict the activities of many of these enzymes (62–64). As well as its involvement in nuclear DNA synthesis, a substantial proportion of PrimPol also localizes to the mitochondria where it is thought to aid in the replication of the small circular mitochondrial genome, in particular by assisting 8-oxo-dG bypass (55,65). PrimPol-knockdown cells exhibit mitochondrial DNA defects (55,65). Similarly, AEPs have been found to be involved in kinetoplast replication in trypanosomes, as discussed below.

The similarity in the damage tolerance activities of archaeal PriS and eukaryotic PrimPol allows inference that PrimPol may have superseded the TLS roles of PriS following its acquisition from large cytoplasmic viruses during the evolution of early eukaryotic organisms (32). Ultimately, this demarcation of roles may have facilitated the specialization of eukaryotic Prim1 as a dedicated replicative RNA primase involved only in primer synthesis during DNA replication, with the acquisition of PrimPol enabling Prim1's TLS activity to become redundant. The discovery of this newly identified AEP and its roles in damage tolerance in eukaryotic organisms again highlights the diversification of roles fulfilled by members of the primase superfamily.

## ESSENTIAL ROLES FOR MULTIPLE AEP ORTHOLOGUES IN TRYPANOSOMES

Kinetoplastids are a group of single-celled protozoa characterized by the presence of kinetoplasts, networks of circular DNA found inside a large single mitochondrion and composed of both maxi-circles (20–40 kb) and mini-circles (0.5–1 kb) (66). Within each organism, this network of kinetoplast DNA must be duplicated prior to division. One particularly well-studied kinetoplastid protozoan is *Trypanosoma brucei*, the causative agent of human African trypanosomiasis (67). Until recently, the kinetoplastid replication machinery of *T. brucei* could not be reconstituted as the primase involved in kinetoplast replication had not yet been identified. Two such primases have since been characterized, PRI1 and PRI2, responsible for maxi-circle and mini-circle replication initiation, respectively (68,69). These primases, like PrimPol, belong to the NCLDV herpesvirus clade of AEPs, and each contains an RRM and a PriCT-2 motif. In each case, RNAi depletion of these enzymes in *T. brucei* causes inhibition of cell growth and the depletion of kinetoplast DNA, clearly suggesting that these AEPs fulfil vital roles in priming kinetoplast DNA replication (68,69).

Recently, two additional PrimPol-like orthologues (PPLs), referred to as PPL1 and PPL2, have been identified in *T. brucei* (58). PPL1 is capable of synthesizing RNA primers up to 50 nt in length on poly(dT) templates. In contrast, PPL2 does not appear to exhibit any primase activity, representing another example where an AEP has ceased to function as a primase (Figure 4). Echoing the TLS activities of PriS and PrimPol, PPL1 and PPL2 also possess damage tolerance synthesis activities, specifically an ability to bypass 6–4 photoproducts and 8-oxo-dG lesions. Perhaps most surprising was the finding that PPL2 is essential for cell survival. Knockdown of PPL2 results in cell cycle arrest following bulk genome duplication. These cells accumulate a lot of DNA damage and die in a pre-mitotic phase. It has been proposed that PPL2 functions as a TLS polymerase that assists in restarting replication downstream of damage or DNA structures during the completion of genome duplication in G_2_ (58). This inability to bypass damage likely leads to the generation of DSBs observed when PPL2 is knocked down. The existence of PPL2 is probably a result of the duplication and subsequent diversification of PPL1 to remedy DNA replication issues specific to trypanosomes and other protists, such as the replication of repetitive sequence elements. These examples again demonstrate how AEPs have diversified to fill a range of roles in both nuclear and mitochondrial DNA metabolism.

## PRIMASES ACTING AS EXTRA-CHROMOSOMAL PLASMID REPLICASES

Plasmid DNA replication is another cellular process where priming of DNA synthesis is required. In addition to PriS, some archaea possess additional AEPs encoded by extra-chromosomal plasmids, which are thought to partake in both the initiation and replication of these small circular plasmids. The first of these to be identified was ORF904 encoded by the pRN1 plasmid (∼5 kb) of *Sulfolobus islandicus*. ORF904 belongs to a novel family of primases present sporadically in crenarchaeal plasmids and Gram-positive bacterial plasmids, the Prim-Pol family (11). The enzyme is composed of an N-terminal AEP domain and a C-terminal helicase/translocase domain. This AEP displays both DNA-dependent RNA/DNA primase and DNA polymerase activity (Figure 4), with the helicase domain exhibiting DNA-dependent adenosine triphosphatase (ATPase) activity (20). Notably, ORF904 shows a preference to generate DNA primers and, in the presence of dNTPs, it can extend these primers by several kilobases (64). The crystal structure of its AEP domain revealed that this enzyme shares strong structural similarities with the *Pyrococcus* archaeal primase (20), particularly in the arrangement of the metal coordinating acidic residues, which display strict conservation within the β-sheet region (Figure 1) (12). Interestingly, both enzymes possess zinc-binding motifs adjacent to the catalytic centre however, somewhat surprisingly, these motifs are located in unrelated positions in each case. This observation has led to the suggestion that the common ancestor of both enzymes did not contain a zinc-binding domain and that two independent insertion events occurred to produce this domain in the evolution of each family (12). In addition to ORF904, a highly related protein has been identified on the pIT3 plasmid of *Sulfolobus solfataricus* called Rep that also comprises an AEP domain fused to a putative helicase (70). The replicative N-terminal domain of this protein, termed Rep245, also possesses dNTP/rNTP-dependent primer synthesis and DNA polymerase activities (Figure 4).

A recent report described the intriguing enzymatic activities of an AEP called PolpTN2 encoded by the pTN2 plasmid of *Thermococcus nautilus*. PolpTN2 is uniquely a fusion of an N-terminal domain homologous to PriS and a C-terminal domain related to PriL (Figure 2) (28). This domain conformation is at odds with other plasmid-encoded primases, which are typically fused to helicases. Nevertheless, similar to other archaeal plasmid-encoded primases, PolpTN2 exhibits primase and DNA polymerase activities. The primase activity of PolpTN2 is exclusively limited to using dNTPs. In addition, the enzyme also has terminal transferase activity, which is greatly enhanced by the removal of the PriL-like region of the protein. This removal also confers reverse transcriptase activity to the primase (28). Interestingly, PolpTN2 and Rep(pIT3) lack a zinc-binding motif present in most other AEPs. The observation that the zinc-binding motifs of each AEP family may have arisen independently suggests that these plasmid-encoded AEPs may represent evolutionarily ancestral AEPs.

Bacteria, like archaea, also harbour extra-chromosomal plasmid DNA. Two decades ago, a Rep protein from the colicin E2 (ColE2) plasmid was found to have DNA primase activity (Figure 4) (71,72). A decade later, it was shown that this primase was in fact a member of the AEP family, distantly related to the archaeal AEPs ORF904 (pRN1) and Rep (pIT3) (11). However, it seems that, unlike the archaeal plasmid AEPs, Rep (ColE2) functions solely as an RNA primase, rather than as a DNA Prim-Pol. This enzyme, in addition to DNA polymerase I, is required for ColE2 DNA replication *in vitro*. Rep (ColE2) binds specifically to the plasmid's origin of replication where it initiates synthesis through the generation of a short RNA primer, allowing DNA polymerase I to subsequently proceed with DNA replication (64). Thus, it appears that Rep (ColE2) functions as a plasmid-specific bacterial primase.

Another bacterial plasmid, RSF1010, found in a broad host range of over Gram-negative and some Gram-positive bacteria, encodes three Rep proteins; RepA, a helicase, RepB’, an AEP primase and RepC, a replication initiator protein (73,74). RSF1010 contains two primase recognition sites *ssiA* and *ssiB*, each of which are recognized by RepB’ allowing the independent synthesis of two primers that can then be extended by the host DNA polymerase III. The crystal structure of RepB’ revealed the presence of two distinct domains; a large N-terminal domain containing two anti-parallel β-sheets flanked by six α-helices and a smaller C-terminal region with a bundle of five α-helices. Notably, the enzyme lacks a zinc-binding motif (Figure 1) (75). This structure reveals a strong structural similarity between the N-terminal domain of RepB’ and the catalytic domain of *P. furiosus* PriS. However, these enzymes share limited sequence homology, in addition to differences in ssDNA template recognition and in their requirements for priming. The structure of the catalytic core of RepB’ bound to a *ssiA* recognition site has provided significant insights into DNA recognition by these primases (Figure 5C) and suggested a mechanism for initiation of plasmid DNA replication (69). Interestingly, RepB’ displays a high degree of thermostability, presumably a result of its structural similarity to primases of the thermophilic archaea, raising interesting questions about the evolutionary origins of the RSF1010 plasmid (75).

The two bacterial plasmid AEPs discussed here, therefore, stand in contrast with those of archaea. The bacterial Rep (ColE2) and RepB’ enzymes represent prototypical AEPs, employed purely in initiating replication through synthesis of a short RNA primer. In contrast, the archaeal plasmid AEPs are proficient Prim-Pols, able to initiate and proceed with bulk replication of their host plasmid DNA. The conservative primase ability and lack of polymerase activity exhibited by these bacterial primases should not, however, be thought typical of all bacterial AEPs. The *Bacillus cereus* genome encodes *Bc*MCM (mini-chromosome maintenance), an AEP/MCM primase/helicase from an integrated prophage. *Bc*MCM was originally identified through BLAST analysis as an MCM homologue, with an N-terminal region of weak homology to AEPs (Figure 2) (76). Initial biochemical studies identified 3′-5′ helicase and ssDNA-stimulated ATPase activity, but also noted the absence of any primase activity (77). However, a more recent structure/function study was able to detect not only helicase activity, but also primase and DNA-dependent DNA polymerase activities (Figure 4) (78). Interestingly, like many archaeal AEPs, *Bc*MCM displays a strong preference for dNTPs during primer synthesis and extension. Together, these findings suggest that BcMCM may act as an important multi-functional enzyme, potentially being deployed in special circumstances during *B. cereus* DNA replication, such as the re-initiation of leading strand replication following fork stalling. Importantly, *Bc*MCM is not the only bacterial AEP with an unconventional cellular role. As discussed previously multifunctional AEPs are also required for DNA DSB, and probably other, repair processes in most bacterial species (16).

## VIRAL AEPs INVOLVED IN DNA REPLICATION

As alluded to earlier, many of the AEPs distributed across the bacterial, archaeal and eukaryotic genomes appear to have viral origins. Indeed, many viruses encode their own AEPs including, UL52-like primases from herpes simplex viruses, D5-like primases from NCLDVs and Lef-1 primases from phage and baculoviruses (11). As is the case for cellular AEPs, viral AEPs also fulfil a number of key roles in DNA metabolism, particularly during replication.

Perhaps the most well studied of the viral AEPs is the UL5-UL8-UL52 heterotrimeric primase-helicase complex found in the herpes simplex virus family (79). Originally identified in herpes simplex virus type 1 (HSV-1), a large double-stranded DNA virus, the UL5-UL8-UL52 complex is encoded by three of the seven genes essential for replication of the HSV-1 chromosome (80). Of these three proteins, UL52 was identified as the AEP responsible for priming DNA replication (80,81), UL5 has helicase activity (82) and UL8 appears to be required for utilization of primers by the UL30/UL42 polymerase. However, UL8 is dispensable for helicase and primase activity of UL5/UL52 (83,84). Where most primases have a zinc-binding motif in their catalytic domains (10,12–13), UL52 has a strand-rich zinc finger domain that is located separately at its C-terminus (Figure 2). This architecture is similar to PrimPol and, likewise, this UL52 zinc finger domain is absolutely required for primase activity *in vivo* (85). The UL52 primase is capable of producing ribonucleotide primers of ∼8–12 nt in length, which are critical for initiating replication of the 153 kb viral genome (80).

Another group of large viruses encoding AEPs are the poxviruses, which includes smallpox, that undertake DNA replication in the cytoplasm of infected cells (86). Most studies of the poxviruses have focussed on vaccinia virus (VACV), which possesses D5, an AEP/helicase fusion protein (Figure 2) (87). This enzyme has a C-terminal domain belonging to the helicase superfamily III and an N-terminal region with sequence and structural features similar to AEPs (87). The N-terminal AEP domain of D5 is essential for viral replication in VACV-infected cells (87). In addition, this enzyme exhibits primase activity *in vitro* and stringent template specificity, strongly suggesting a priming role for this enzyme in VACV DNA replication (Figure 4) (87,88). Based on extensive *in silico* analysis, Iyer *et al*. grouped the D5-like primases of poxviruses, irdoviruses, mimivirus and African swine fever virus with the herpes simplex virus primases and their eukaryotic homologues, including eukaryotic PrimPol (11). These enzymes, in addition to the A468R-like proteins of phycodnaviruses, make up the NCLDV-herpesvirus clade of AEPs. However, it should be noted that not all viral AEPs belong to this primase clade.

Unlike the UL52 herpesvirus and D5-like poxvirus AEPs, Lef-1-like primases of baculoviruses represent a family of AEPs that are more closely related to the replicative and NHEJ AEPs that collectively form the AEP-proper clade (11). Strikingly, the Lef-1-like primases of baculoviruses have the capacity to synthesize RNA primers that are extended by up to several kilobases in length (89) (Figure 4). This ability is in line with the extension activities of the archaeal replicative primase PriS from *Pyrococcus*, supporting the fact that these enzymes belong to the same AEP clade. However, it has been suggested that the extension capabilities of Lef-1-like primases may be limited by other replication factors *in vivo* (89). Nevertheless, this ability raises the possibility that these enzymes may play additional roles in primer extension.

In contrast to the RNA primase activities of the viral AEPs discussed above, the gp43-like proteins of corynephage BFK20, do not share this rNTP incorporation preference. Instead, the gp43-like proteins, part of the Prim-Pol clade of AEPs that includes ORF904 and Rep(pIT3), can only incorporate dNTPs (90). In addition, the gp43-like proteins, similar to the archaeal AEPs, display both DNA primase and polymerase activities (90). Thus, showing that even within viruses, AEPs form a diverse group of enzymes with varying catalytic capabilities and potentially divergent roles.

## REPRIMING DNA SYNTHESIS FOLLOWING REPLICATION STALLING

Although much of this review has focused on the novel roles of primases in DNA metabolism, particularly the ability of many AEPs to also act as template-dependent polymerases, we would also like to highlight the novel deployment of repriming mechanisms. It was first appreciated by Rupp and Howard-Flanders that gaps or discontinuities are produced during the replication of DNA in *E. coli* exposed to significant levels of UV damage, that allow genome synthesis to proceed at near normal rates (91). This maintenance of ‘normal’ replication fork rates is largely achieved by repriming of DNA synthesis by a DnaG-dependent complex post-lesion thus preventing excessive slowing of genome synthesis (92,93). It has been proposed that a similar mechanism might also exist in eukaryotes but until recently the enzyme responsible for this restart mechanism remained unidentified.

It has now been reported that PrimPol is likely employed in this re-priming mechanism (56,57). Importantly, the dispensability of the zinc finger (ZnF) domain of PrimPol for polymerase and TLS activities, coupled with its strict requirement for primase activity, has allowed separation of function studies to be performed *in vivo*. These studies revealed that an intact ZnF domain is required for PrimPol to maintain normal replication fork rates following UV damage, suggesting that the primase activity of the enzyme is necessary for replication restart. Notably, an intact ZnF is not required to maintain replication fork speeds during unperturbed replication but it remains possible that the ZnF domain is required for recruitment or efficient TLS *in vivo* (56). Nevertheless, the involvement of PrimPol in re-priming DNA replication post-damage appears to be the most likely explanation for the observed phenotypes (56,57). Evidence supporting this includes studies in DT40 cells demonstrating that fork progression rates are largely unaffected in the absence of many TLS components, with the exception of REV1 (94). In addition, in mammalian cells UV irradiation of cells defective in Pol η did not cause persistent fork stalling but did lead to the generation of ssDNA gaps in replicated DNA (95). These studies suggest that re-priming occurs, likely facilitated by PrimPol, during replication, leaving behind ssDNA gaps opposite the lesions that are subsequently filled in by TLS in a post-replicative manner. Importantly, the potential involvement of PrimPol in re-priming DNA replication begs the question as to why the replicative primase Prim1 cannot also be employed in this role? It seems likely that, at least for lagging strand synthesis, this will be the case. Here, re-priming would simply require the initiation of a new Okazaki fragment downstream of the lesion, facilitated by Prim1-dependent re-priming. However, the requirement for PrimPol to re-prime synthesis on the leading strand may be of more importance due to its preference to utilize dNTPs during primer synthesis thus preventing incorporation of RNA. This offers a distinct advantage over Prim1 as it eliminates the possibility of RNA processing or hydroysis that would lead to formation of breaks on the leading strand that would eventually result in potentially lethal DSBs.

## WHY DO PRIMASES PREFER TO INCORPORATE RNA INTO DNA?

A common feature of many AEP enzymes, including replicative primases, is their marked preference to incorporate NTPs, rather than dNTPs, into the synthesized strand. A pertinent question here is why these specialized polymerases have maintained this preference to prime replication or repair damaged DNA by synthesizing RNA, which is much less stable due to the presence of a 2′ OH moiety that makes the sugar much more prone to hydrolysis. This is particularly surprising in the case of mammalian DNA replication, where the replicative primases incorporate lots of RNA into the newly synthesized DNA that must then be excised and replaced before genome duplication is completed.

A shared structural feature of all AEP-related primases is their open and malleable active sites that, unlike canonical polymerases, enables them to accommodate a wide variety of DNA configurations including: ss/ds DNA, mismatches, lesions and even termini of DSBs (Figures 1 and 5). However, the price to be paid for this catalytic flexibility is low fidelity. To illustrate this point in more detail let us examine the NHEJ AEPs. These enzymes are highly adaptive polymerases that have effectively lost their primase activity and evolved to accommodate a wide range of DNA configurations in their active sites and perform an extensive variety of extension activities to ensure that DSB are repaired, irrespective of the nature of the break. However, they also preferentially incorporate NTPs to fill in any gaps with RNA, which are then preferentially ligated to seal the breaks (48). Why do these enzymes prefer to fill the gaps with RNA instead of DNA? The likely explanation for this preference is because of the very low fidelity exhibited by these polymerases. By incorporating RNA into the repaired breaks, the enzyme is ‘flagging up’ the bases that it has incorporated. Once DSB repair has been completed by NHEJ, ribonucleases (e.g. RNase H2) can then excise the RNA, which can then be replaced with DNA by more accurate patch repair polymerases. In the case of DNA replication, primer synthesis is also an highly inaccurate process and given the large number of regions where RNA is incorporated into the genome, particularly on the lagging strand, this would result in the introduction of a very high mutagenic load during every round of replication. To prevent this from occurring in cells it is likely that, as during NHEJ, RNA is preferred for primer synthesis to demarcate regions of low fidelity synthesis that are subsequently excised and replaced with DNA in a more faithful synthesis process that occurs before the completion of genome synthesis.

## CONCLUDING REMARKS

**Figure 6.**
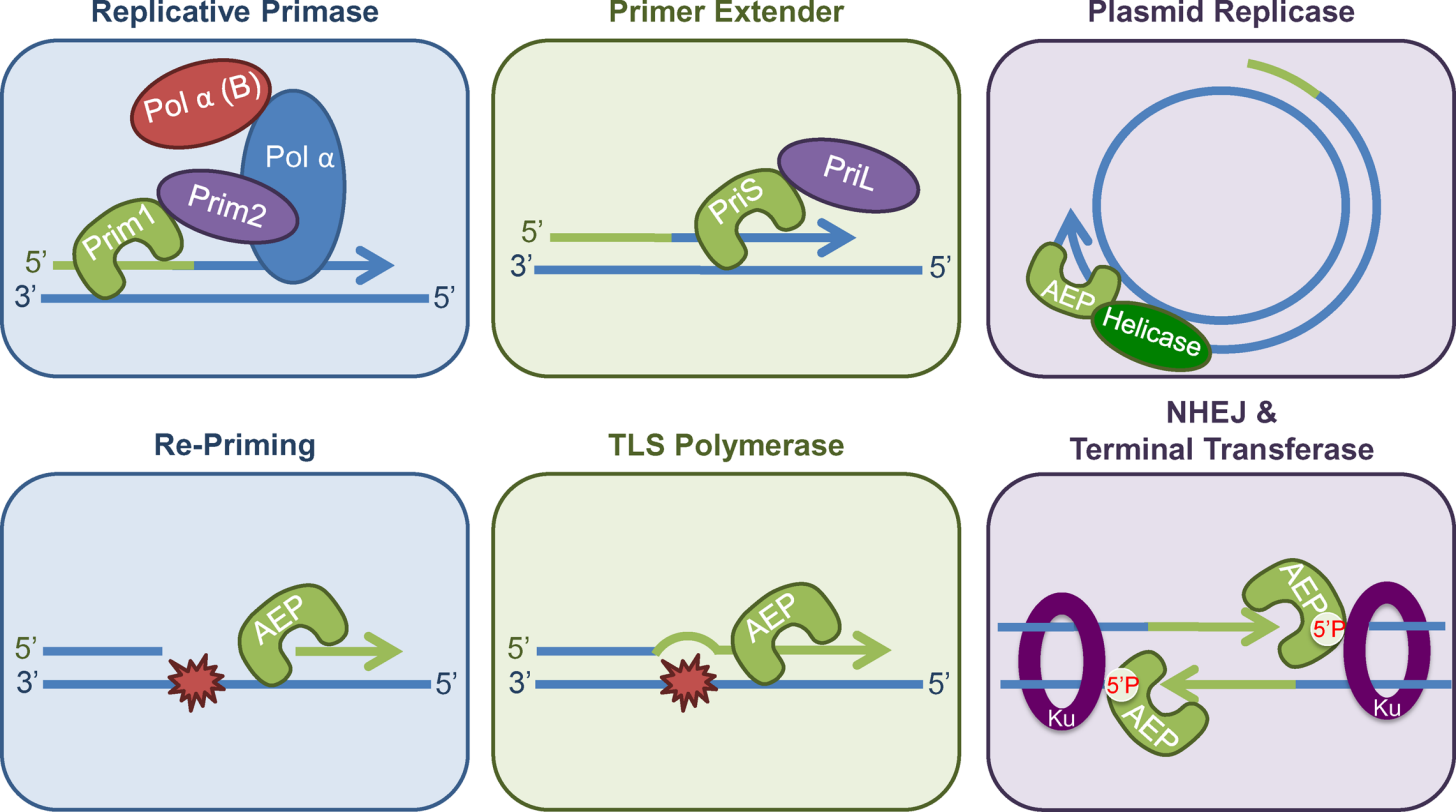
Diversity of functional roles fulfilled by AEPs. AEP superfamily members are employed in many different biological roles, in addition to replicative primases. These enzymes are also utilized as primer extenders, plasmid replicases, damage-tolerance re-priming enzymes, TLS polymerases, NHEJ DNA break repair and terminal transferase polymerases.

Since the first discovery of DNA replicative polymerases, well over half a century ago, it has been fully appreciated that there are also a more diverse range of families within this general classification, giving rise to many additional enzymes that have distinct roles in a wide variety of nucleic acid metabolism processes in cells, from replication to transcription. In contrast, the possibility that DNA primases may also have additional members and roles in cells has largely been overlooked until relatively recently. The reasons for this are partially down to their name, which categorically assigns a sole function to the bespoke nucleotidyltransferase activity associated with the first identified replicative primases, thus deterring significant further investigation of additional activities and functions associated with this grouping of enzymes. Whilst this primase label appropriately describes the *de novo* synthesis of RNA primers associated with toprim-related DnaG primases and a small subset of the AEP-proper clade (Prim1), in most cases it is a misnomer that does not adequately describe the function of the vast majority of members that make up this superfamily (Figure 6). AEP primases and canonical DNA polymerases most likely evolved from a common ancestral nucleic acid recognition domain (11), the RRM and share a number of common catalytic features, including divalent metal-dependent catalyzed extension of nucleic acids in a 5′-3′ direction. We therefore propose that all members of the AEP primase superfamily should be reclassified as belonging to the broad general grouping of enzymes called polymerases and, within this umbrella term, be further sub-classified as belonging to a sub-grouping of enzymes called Prim-Pols to reflect their dual origins and, in most cases, their capacity to perform both synthesis functions (Figure 4). This term has already gained acceptance to describe a number of different microbial and eukaryotic AEPs involved in a diverse range of functions, including plasmid replication, lesion bypass and repriming.

In this Survey and Summary, we have described the current landscape of AEP-related members and functions found throughout the major domains of life that supports this conclusion. Although many additional roles have been identified for Prim-Pols over the last decade or so (Figure 6), much more remains to be discovered about the diverse functions and pathways in which these highly adaptable enzymes operate. For example, given that they can perform both priming and template-dependent synthesis events, it is likely that Prim-Pols undertake roles in other key cellular pathways, such as restart and bypass mechanisms associated with replication fork stalling at structural impediments. Given the propensity of many Prim-Pols to incorporate RNA during synthesis, it is also likely that they may also be involved in a range of transcriptional-related processes. AEP members are associated with some CRISPR operons suggesting potential roles in other processes, such as viral ‘immunity’ in microbes. These are just some potential examples of novel AEP functions and many more probably await to be discovered.

In addition to understanding the myriad of different functions associated with Prim-Pols, the identification of novel AEPs also provides an opportunity to explore potential associations between these enzymes and human disease. For example, a mutation of human PrimPol has been identified in individuals with high myopia and is associated with defective DNA replication, significantly reducing its polymerase activity (96). PrimPol mutations have also been identified in human cancers (97–101) and it is over-expressed in others (102), suggesting possible roles in overcoming replication stress in disease tissues. As PrimPol appears to play important roles in maintaining replication fork progression, but is not an essential mammalian enzyme, developing PrimPol inhibitors that disrupt DNA synthesis may be a possible strategy to treat a range of conditions. Many Prim-Pols are also important for the life cycles of many pathogenic bacteria, viruses and protozoa that infect mammals and therefore these enzymes are also potentially attractive targets for the development of novel anti-microbial agents that specifically target essential DNA replication and repair pathways in these organisms.
